# Survey data on perceived sustainability and revisit intention of tourists to community-based tourism

**DOI:** 10.1016/j.dib.2025.111773

**Published:** 2025-06-11

**Authors:** Kien Trung Dao, Dat Dinh Nguyen, Duy Van Nguyen, Dat Ngoc Nguyen, Huong Thi Lan Pham

**Affiliations:** a Phenikaa University, Hanoi, Vietnam; b Foreign Trade University, Hanoi, Vietnam; c QAglobal, Hanoi, Vietnam

**Keywords:** Sustainable tourism, Community-based tourism, Perceived value, Destination image, Revisit intention

## Abstract

Tourism in mountainous provinces is attracting everyone in Vietnam. This kind of tourism is becoming popular in this country. Additionally, Sustainable tourism is increasingly of interest to tourists as well as organizations. The purpose of this study is to examine how tourists' perception of sustainability impacts their perception of and intentions to revisit community-based tourism destinations. The authors collected data on tourists' perceived sustainability and intentions to revisit community-based tourism sites. The survey data was collected from 295 tourists visiting community-based tourism destinations in mountainous provinces of Vietnam. We assessed the reliability using Confirmatory Factor Analysis (CFA) and discriminant validity using the AMOS software. The data will contribute insights into the research area of perceived sustainability and revisit intentions at community-based tourism destinations. Besides, the role of destination image, perceived value, and satisfaction are considered in this study.

Specifications Table

**Table:** 

Subject	Social Sciences (General)
Specific subject area	Sustainable tourism
Type of data	Table, Raw, analyzed
Data collection	Survey forms are provided to the homestay host for distribution to guests by convenience method. The survey is collected from 5/2022 to 07/2022 via direct survey and resulted in 295 responses.
Data source location	Region: Asia Country: Vietnam
Data accessibility	The dataset is provided as a supplementary file. Direct URT to data: https://data.mendeley.com/datasets/7gcj5wwrbz/1 Data identification number DOI: 10.17632/7gcj5wwrbz.1

## 1 Value of the Data

• The data can be usefully used for general business statistical practices. For example, using SEM to test the relationship between perceived sustainability and revisit intention, t-test or ANOVA to explore the difference among demographic factors and perceived sustainability and revisit intention, etc.
• The data offers evidence for stakeholders of destinations (residents, businesses, government agencies) to undertake sustainable development activities to attract tourists
• The data is able to use to comparisons with other countries. Combining with studies in other countries will help researchers make discoveries about these national differences.

## 2 Background

Tourism indeed plays an essential role in the economy and is also considered an effective method of reducing poverty in traditional communities [1]. It is estimated that tourism occupies around 10 % of the GDP globally and creates about 11 % of direct and indirect jobs globally [2]. In Vietnam, the natural and cultural heritage makes tourism a significant part of economic development. In 2019, tourism achieved total revenue of approximately $31.4 million, contributing nearly 8 % to the overall GDP. The tourism industry maintains a growth rate of over 10 % annually and creates millions of jobs [2]. Tourism reduces poverty effectively and improves the local people's living standards by providing different jobs other than traditional ones, or selling the local products [3], creating employment opportunities, improving the infrastructure, preserving the cultural values, and encouraging cultural exchange.

Community-based tourism (CBT) was considered one of the sustainable tourism development methods [4]. It has been introduced in many parts of the world because it seeks to achieve economically, environmentally, ad socio-culturally sustainable development [5]. Various model of CBT has been expressing their ability in improving local economic status, such as rural community, fishing communities, the residents of small islands, and aboriginal communities [6]. The development of the CBT also increases the number of infrastructure systems such as roads, parks, and entertainment spots noticeably, reverses and restores cultural festivals, and benefits the residents [7]. Furthermore, tourism may allow residents to appreciate and respect their own local culture and social structure, thereby increasing the sustainability of the social-ecological system. In minority communities, residents revitalized the indigenous culture to perform it to tourists [3]. Furthermore, with abundant natural resources, topography, beautiful landscapes, and unique flora and fauna species, tourism promotes the residents of environmental protection to provide high-quality recreation experiences for tourists [3]. Therefore, community-based tourism plays a vital role in poverty reduction and fostering community development, supporting community sustainability.

In Vietnam, community-based tourism emerged later due to post-Vietnam War challenges, gaining momentum in the late 1990s in provinces like Ban Lac and Ta Van. Officially recognized in 2017′s Tourism Law, its growth was supported by the EU-funded ESRT program (2011-2016), resulting in 300+ sites and 5,000 homestays hosting 100,000 tourists by 2020 [8]. This approach empowers marginalized communities, preserves cultural heritage, and drives sustainable economic growth [7]. Community-based tourism enhances local livelihoods, fosters income generation, and bolsters infrastructure. Diverse models ensure sustainable growth by valuing and safeguarding local cultures, raising awareness among communities, and promoting both traditions and development. A survey within the ESRT program revealed substantial tourist interest in environmentally friendly, culturally immersive, and locally supportive experiences, signaling a strong demand for responsible and sustainable community-based tourism in Vietnam's mountainous areas, benefiting cultural and economic aspects alike.

The purpose of this study gathers survey data to assess the factors which related to perceived sustainability as well as tourists' perceptions and revisit intentions at community-based tourism destinations.

## 3 Data Description

The survey participants were identified as tourists who had a minimum of two days of experience at the selected destinations. We contacted the homestay hosts to request their assistance in participating in the survey. The questionnaire and informed consent forms were printed and sent to the hosts who agreed to support our survey. An interview guide and an explanation of the research purpose were also provided to the hosts. The hosts conducted the survey by gathering the opinions of the staying tourists at suitable times, typically around the check-out time of the homestays. The hosts introduced the survey and invited the guests to participate. Those who agreed to participate in the study received a printed questionnaire and an informed consent form to sign and indicate their willingness to participate. The items and questionnaire are presented in Table 3, Table 4. The informed consent form is in appendix A, and the translated printed questionnaire is in Appendix B. The survey was conducted at convenient times for the tourists, usually before the check-out time of their homestay. As a result, we distributed 400 questionnaires and gained 295 valid questionnaires (105 surveys were not completed and were returned to the homestay host). The rate of valid respondents is 74 % (Table 1).

**Table 1:** Respondents’ profiles (*n* = 295).

Categorical		Number of respondents ( %)
Gender	Male	147(49.8 %)
	Female	148(50.2 %)
Age	< 20	30(10.2 %)
	20–25	58(19.7 %)
	26–30	78(26.4 %)
	31–35	70(23.7 %)
	> 35	59(20.0 %)
Education	High school	80(27.1 %)
	Bachelor/Engineer	136(46.1 %)
	Master	79(26.8 %)

In addition to survey information related to the survey objects, this article also provides information about the factors the perceived sustainability, Revisit intention, Destination image, Perceived value and satisfaction. The level of tourists' assessment of these factors is also presented to provide information about the current situation related to this tourism activity. Accordingly, all factors are evaluated at good level (mean of score ranges from 3.83 to 4.06 on a 5-point Likert scale). Details are presented in table 2.

**Table 2:** Describes visitors' assessments of factors related to perceived sustainability and revisit intention

Factors	Minimum	Maximum	Mean	Std. Deviation
ECO	1	5	3.83	.738
CUL	1	5	4.06	.710
ENV	1	5	3.86	.807
IMG	2	5	4.02	.616
VAL	1	5	3.96	.728
SAT	1	5	4.03	.758
REV	1	5	4.01	.755

**n=295**				

VAL = perceived value, IMG = destination image, SAT = satisfaction, SUS- perceived sustainability included: ECO: economic sustainability; CUL: socio-cultural sustainability and ENV: environmental sustainability, REV = revisit intention./.

## 4 Experimental Design, Materials and Methods

To measure the constructs in the model, we refer to literature and previous studies [9]. Specifically, the perceived sustainability construct was measured by a multidimensional scale comprising three components (1) economic sustainability, (2) socio-cultural sustainability, and (3) environmental sustainability, with fourteen items adapted from Iniesta-Bonillo et al.[9]. The perceived value construct was evaluated by four items based upon Sun et al.[10] and Iniesta – Bonillo et al.[9]. The satisfaction construct was measured by four items referenced from Iniesta – Bonillo et al. [9]. The destination image construct was assessed by five items adapted from Sun et al.[10]. Revisit intentions construct was evaluated by four items referenced from Sun et al.[10]. The items were translated from English to Vietnamese, and the back translation method was used to keep the original meaning. The data was distributed to visitors at Ban Lac, Mai Chau, Hoa Binh. The Likert scale five-point was used to evaluate each item in the construct with 1 – totally disagree and 5 – totally agree. The survey was conducted from May 2022 to July 2022. The research project was also approved by the scientific ethics committee at the Quantitative Research Center of QA Global company.

We used the multivariate data analysis method to analyze the data surveyed. The model constructs were evaluated for reliability, validity, and fit to the actual data by confirmatory factor analysis (CFA). The criteria chosen for CFA analysis include Chi-square/df being less than 3, CFI, TLI, and IFI being greater than 0.9, and RMSEA being less than 0.08 [11]. The factor loadings of each item in the constructs are greater than 0.5, presenting that the constructs in the model reach convergent validity [11]. To the discriminant validity of each construct, we use the 95 % confidence interval of correlation among the constructs. If the 95 % confidence interval of correlation does not contain one value, the constructs achieve discriminant validity. To evaluate the reliability, we use Cronbach's alpha, and composite reliability coefficients with criteria are greater than 0.7, and the average variance extracted is greater than 0.5.

The analysis result indicated the constructs achieved overall fit with the actual data: Chi-square/df = 2.092 was less than 3, CFI = 0.919; TLI = 0.908; IFI = 0.920 were greater than 0.9, and RMSEA = 0.061 was less than 0.08. The factor loadings of the items in each construct are all greater than 0.5. Therefore, we could conclude the items that measure the constructs in the model reached convergent validity. The Cronbach's alpha and composite reliability coefficients were all greater than 0.7. The average variance extracted from each construct was greater than 0.5, showing that the constructs in the model were reliable (Table 3, Table 4).

**Table 3:** Convergent validity and reliability test of each sub-construct in the perceived sustainability scale

Code	Statement	Factor loadings	Cronbach's Alpha	Composite reliability	Average variance extracted
***Perceived sustainability***					
*Economic sustainability*					
ECO1	The tourism destination is invested in attracting tourists (amusement parks, shopping centers, etc.).	0.743	0.769	0.763	0.518
ECO2	The destination is equipped with good infrastructure (roads, vehicles ...)	0.744			
ECO3	The destination's service is worth the tourist's expenditure.	0.671			
ECO4	I think the investment cost in the destination is not as much as the investors' benefits.	Remove			
ECO5	I see that promoting local tourism provides many benefits for the people.	Remove			
*Cultural sustainability*					
CUL1	I think the heritages in the tourist destinations are well-preserved such as historical or cultural sites.	0.713	0.806	0.808	0.513
CUL2	I think the cultural heritage values are well-preserved, such as traditions, festivals, or traditional cultural activities.	0.759			
CUL3	I think the cultural heritage and the value of local history are well-preserved by the tourism activities at the destination.	0.697			
CUL4	Overall, I think the tourist destination preserves its cultural values ​​effectively.	0.694			
*Environmental sustainability*					
ENV1	I think the waste pollution at the tourist destination is controlled/ maintained at an acceptable level.	0.788	0.85	0.856	0.599
ENV2	I think the air pollution at the tourism destination is controlled/ maintained at an acceptable level.	0.824			
ENV3	I think the water pollution at the tourism destination is controlled/ maintained at an acceptable level.	0.786			
ENV4	I think the noise pollution at the tourism destination is controlled/ maintained at an acceptable level.	Remove			
ENV5	I think the density of tourists at the destination is maintained appropriately in the peak season.	Remove			
ENV6	Overall, I think that environmental protection activities are promoted strongly.	0.692

**Table 4:** Reliability and convergent validity of each construct

Code	Statement	Factor loadings	Cronbach's Alpha	Composite reliability	Average variance extracted
***Perceived sustainability***					
ECO	*Economic sustainability*	0.918	-	0.827	0.623
CUL	*Cultural sustainability*	0.833			
ENV	*Environmental sustainability*	0.577			
***Destination image***					
IMG1	The tourism conditions can support the tourist activities such as infrastructure, accommodation, restaurants, or amusement parks).	Remove	0.735	0.68	0.516
IMG2	I had an interesting experience at the tourist destination.	0.699			
IMG3	I underwent the experience of being involved in the tourism community.	Remove			
IMG4	I found opportunities to explore local cultural activities.	Remove			
IMG5	The tourist destination creates the most invaluable experience I have ever had before.	0.737			
***Perceived value***					
VAL1	I experienced the excellent quality of service in the tourism destination.	0.658	0.727	0.782	0.547
VAL2	The service cost at the destination is appropriate for you.	0.805			
VAL3	You think that tourist destination is the worth choice.	0.748			
VAL4	Overall, it is worthwhile for you to experience the tourist destination.	Remove			
***Satisfaction***					
SAT1	The trip met my expectation.	0.795	0.85	0.875	0.6
SAT2	I enjoy this trip.	0.777			
SAT3	The trip is significant for me.	0.811			
SAT4	Overall, I feel satisfied with this tourist destination.	0.713			
SAT5	I am willing to pay a surcharge to support the conservation and sustainable tourism development at this destination.	Remove			
***Revisit intention***					
REV1	I intend to return to this tourist destination in the future.	Remove	0.84	0.81	0.516
REV2	I desire to come back to this destination in the future.	0.716			
REV3	I will recommend this destination to others.	0.773			
REV4	I will tell the positive factors about this destination to others.	0.716			
REV5	I will encourage my friends to visit this destination.	0.667			

The analysis result by bootstrapping method to estimate a 95 % confidence interval (95 %CI) of the correlation among constructs in the model, indicating the 95 % CI of correlation coefficients did not contain one value (Table 5). It is shown that the constructs in the model achieved discriminant validity.

**Table 5:** Discriminant validity of the constructs

Constructs	VAL	IMG	SAT	SUS	REV
VAL	1				
IMG	0.851 (0.745-0.975)	1			
SAT	0.932 (0.867-0.997)	0.819 (0.654-0.973)	1		
SUS	0.722 (0.595-0.835)	0.769 (0.626-0.896)	0.694 (0.569-0.799)	1	
REV	0.905 (0.806–0.995)	0.746 (0.578–0.913)	0.936 (0.874–0.998)	0.654 (0.521–0.771)	1

*Notes:* The values in parentheses are the 95 % confidence intervals of the correlation coefficients. VAL, perceived value; IMG, destination image; SAT, satisfaction; SUS, perceived sustainability; REV, revisit intention.

## Limitations

Although the data presented a variety of information related to key factors related to perceived sustainability and revisiting the intention of tourists to community-based tourism. However, this study used non-probability sampling method. This data collection method is not guaranteed to accurately control the representativeness of individual characteristics within the tourist population. Therefore, the probability sampling method can be considered for further studies. The probability sampling method will help better classify specific characteristics similar to the entire population, such as gender, education, and age. This method allows for better control over the sample's representativeness of the tourist population. Additionally, the study only surveyed domestic tourists and did not consider foreign tourists. The characteristics of tourists from different countries may provide more information for future analysis. Therefore, future survey data on foreign tourists is needed.

## Ethics Statement

Respondents to questionnaire participated voluntarily. Also, all personal information such as name, identity is not collected to ensure the respondent's privacy.

## CRediT Author Statement

**Kien Trung Dao**: Methodology, Data curation, Writing- Reviewing and Editing. **Dat Dinh Nguyen:** Conceptualization, Writing- Original draft preparation, Visualization, Investigation. **Duy Van Nguyen**: Data analysis, Writing- Original draft preparation. **Dat Ngoc Nguyen**: Writing- Original draft preparation. **Huong Thi Lan Pham**: Writing- Original draft preparation.

## Data Availability

Mendeley DataSurvey data on perceived sustainability and revisit intention of tourists to community-based tourism (Original data).

## Appendix Supplementary materials

Image, application 1

Image, application 2

Image, application 3
