# Supplementary material for: Survey data on perceived sustainability and revisit intention of tourists to community-based tourism
Source: Data Brief. 2025 Jun 11;61:111773. doi: 10.1016/j.dib.2025.111773 (PMC12221664; doi:10.1016/j.dib.2025.111773)
Supplement: Supplementary file 1 [file mmc1.doc]

**Questionnaire**

**Question content**

Below are the opinions about the tourist destination you are experiencing. Please circle the corresponding answers to your level of agreement with those statements (the higher the score, the more agreement). In which

| **1. Totally disagree** |  | **5. Totally agree** |
| --- | --- | --- |

| **Code** | **Contents** | Level of agreement | | | | |
| --- | --- | --- | --- | --- | --- | --- |
| ***Perceived sustainability*** | | | | | | |
| *Economic sustainability* | | | | | | |
| ECO1 | The tourism destination is invested in attracting tourists (amusement parks, shopping centers, etc.). | 1 | 2 | 3 | 4 | 5 |
| ECO2 | The destination is equipped with good infrastructure (roads, vehicles ...) | 1 | 2 | 3 | 4 | 5 |
| ECO3 | The destination's service is worth the tourist's expenditure. | 1 | 2 | 3 | 4 | 5 |
| ECO4 | I think the investment cost in the destination is not as much as the investors' benefits. | 1 | 2 | 3 | 4 | 5 |
| ECO5 | I see that promoting local tourism provides many benefits for the people. |  |  |  |  |  |
| *Cultural sustainability* | | | | | | |
| CUL1 | I think the heritages in the tourist destinations are well-preserved such as historical or cultural sites. | 1 | 2 | 3 | 4 | 5 |
| CUL2 | I think the cultural heritage values are well-preserved, such as traditions, festivals, or traditional cultural activities. | 1 | 2 | 3 | 4 | 5 |
| CUL3 | I think the cultural heritage and the value of local history are well-preserved by the tourism activities at the destination. | 1 | 2 | 3 | 4 | 5 |
| CUL4 | Overall, I think the tourist destination preserves its cultural values ​​effectively. | 1 | 2 | 3 | 4 | 5 |
| *Environmental sustainability* | | | | | | |
| ENV1 | I think the waste pollution at the tourist destination is controlled/ maintained at an acceptable level. | 1 | 2 | 3 | 4 | 5 |
| ENV2 | I think the air pollution at the tourism destination is controlled/ maintained at an acceptable level. | 1 | 2 | 3 | 4 | 5 |
| ENV3 | I think the water pollution at the tourism destination is controlled/ maintained at an acceptable level. | 1 | 2 | 3 | 4 | 5 |
| ENV4 | I think the noise pollution at the tourism destination is controlled/ maintained at an acceptable level. | 1 | 2 | 3 | 4 | 5 |
| ENV5 | I think the density of tourists at the destination is maintained appropriately in the peak season. |  |  |  |  |  |
| ENV6 | Overall, I think that environmental protection activities are promoted strongly. |  |  |  |  |  |
| ***Destination Image*** | | | | | | |
| IMG1 | The tourism conditions can support the tourist activities such as infrastructure, accommodation, restaurants, or amusement parks). | 1 | 2 | 3 | 4 | 5 |
| IMG2 | I had an interesting experience at the tourist destination. | 1 | 2 | 3 | 4 | 5 |
| IMG3 | I underwent the experience of being involved in the tourism community. | 1 | 2 | 3 | 4 | 5 |
| IMG4 | I found opportunities to explore local cultural activities. | 1 | 2 | 3 | 4 | 5 |
| IMG5 | The tourist destination creates the most invaluable experience I have ever had before. | 1 | 2 | 3 | 4 | 5 |
| ***Perceived value*** | | | | | | |
| VAL1 | I experienced the excellent quality of service in the tourism destination. | 1 | 2 | 3 | 4 | 5 |
| VAL2 | The service cost at the destination is appropriate for you. | 1 | 2 | 3 | 4 | 5 |
| VAL3 | You think that tourist destination is the worth choice. | 1 | 2 | 3 | 4 | 5 |
| VAL4 | Overall, it is worthwhile for you to experience the tourist destination. | 1 | 2 | 3 | 4 | 5 |
| ***Satisfaction*** | | | | | | |
| SAT1 | The trip met my expectation. | 1 | 2 | 3 | 4 | 5 |
| SAT2 | I enjoy this trip. | 1 | 2 | 3 | 4 | 5 |
| SAT3 | The trip is significant for me. | 1 | 2 | 3 | 4 | 5 |
| SAT4 | Overall, I feel satisfied with this tourist destination. | 1 | 2 | 3 | 4 | 5 |
| SAT5 | I am willing to pay a surcharge to support the conservation and sustainable tourism development at this destination. | 1 | 2 | 3 | 4 | 5 |
| ***Revisit intention*** | | | | | | |
| REV1 | I intend to return to this tourist destination in the future. | 1 | 2 | 3 | 4 | 5 |
| REV2 | I desire to come back to this destination in the future. | 1 | 2 | 3 | 4 | 5 |
| REV3 | I will recommend this destination to others. | 1 | 2 | 3 | 4 | 5 |
| REV4 | I will tell the positive factors about this destination to others. | 1 | 2 | 3 | 4 | 5 |
| REV5 | I will encourage my friends to visit this destination. | 1 | 2 | 3 | 4 | 5 |

**Personal Information**

Please mark the appropriate information for the following questions:

- Gender: **a.** Male ; **b**.Female
- Age group: **a.** < 20; **b.** 20 - 25; **c.** 25 - 30; **d**. 30 - 35; **e.** > 35
- Education: **a.** High school**; b.** Bachelor/Engineer; **c.** Master

THANK YOU VERY MUCH FOR YOUR HELP
