# Supplementary material for: Survey data on perceived sustainability and revisit intention of tourists to community-based tourism
Source: Data Brief. 2025 Jun 11;61:111773. doi: 10.1016/j.dib.2025.111773 (PMC12221664; doi:10.1016/j.dib.2025.111773)
Supplement: Supplementary file 2 [file mmc2.docx]

**APPENDIX A**

**INFORMED CONSENT FORM FOR PARTICIPATION**

Study Title: Survey data on perceived sustainability and revisit intention of tourists to community-based tourism.
Principal Investigator: Dat Dinh Nguyen
Institution: Foreign Trade University, Hanoi, Vietnam
Contact Information: datnd@ftu.edu.vn

**Purpose of the Study**

You are invited to participate in a research study aimed at assessing the factors which related to perceived sustainability as well as tourists' perceptions and revisit intentions at community-based tourism destinations. The purpose of this survey is to gather information that will contribute to offers evidence for stakeholders of destinations to undertake sustainable development activities to attract tourists

**Study Procedures**

To complete this study, we need your help by answering your opinions about the current tourist destination you are visiting/experiencing. Your opinions are helpful to our research, and there is no right or wrong answer. Therefore, we hope you can spare some time to answer us honestly. All personal information (if any) will be kept confidential and processed through statistical methods that will not appear in the study. If you agree to participate in this study, you will be asked to complete a survey. The survey will take approximately 5-10 minutes to complete. Your participation is voluntary, and you may skip any questions that you do not wish to answer.

**Confidentiality**

Your responses will be kept confidential and will only be used for research purposes. All data collected will be stored securely, and your identity will not be disclosed in any reports or publications arising from this research. Your responses will be anonymized and grouped with others to ensure your privacy.

**Voluntary Participation**

Participation in this survey is entirely voluntary. You may choose not to participate or withdraw from the study at any time without any consequences. If you decide to withdraw, any data collected up until that point will be discarded and not included in the final analysis.

**Potential Risks and Benefits**

There are no known significant risks associated with participating in this study

**Consent**

By signing below, you acknowledge that you have read and understood the information provided above and agree to participate in this survey voluntarily. You understand that you are free to withdraw at any time without any consequences.

**Participant's Name (Printed): ___________________________**

**Participant's Signature: _________________________________**

Thank you for your participation!
